# Editorial: Microbial Biominerals: Toward New Functions and Resource Recovery

**DOI:** 10.3389/fmicb.2021.796374

**Published:** 2021-11-29

**Authors:** Lucian C. Staicu, Eric D. van Hullebusch, Christopher Ackerson

**Affiliations:** ^1^Faculty of Biology, University of Warsaw, Warsaw, Poland; ^2^Université de Paris, Institut de Physique du Globe de Paris, CNRS, Paris, France; ^3^Chemistry Department, Colorado State University, Fort Collins, CO, United States

**Keywords:** microbial biominerals, biomineralization, metals, environmental depollution, resource recovery

Microbes form biominerals via biologically-controlled mineralization (BCM) and biologically-induced mineralization (BIM) (Konhauser and Riding, 2012). BCM is commonly an intracellular process, where microbes employ genetic determinants and enzymes to induce mineralization. The end product (the biomineral) of BCM serves a biological function for its host. Some notable examples include magnetotactic bacteria (the magnetite chain helps target microaerophilic environments) and bacteria that biomineralize carbonates (intracellular carbonate contributes to buoyant density) (Uebe and Schüler, 2016; Görgen et al., 2021). Conversely, mineral formation in BIM does not have a regulatory control and the biomineralization product is generally located outside the cell. Numerous minerals are being formed via this process such as BaSO_4_, PbS or iron minerals. BIM-produced biominerals do not often have a clear biological function. For instance, respiratory-sourced biogenic Se^0^ may contribute to the buoyant density of sludge granules in upflow bioreactors (Staicu and Barton, 2021). However, with the renewed interest in microbial biominerals new biological functions may be acknowledged in the future.

Various industrial effluents are characterized by an abundance of metals/metalloids and anionic complexes present in dissolved and harmful form for aquatic and terrestrial ecosystems. In this context, removing some of these components in the form of stable (bio)minerals is regarded as a sound depollution strategy (Staicu et al., 2021). Because certain bacteria have enzymatic systems with high affinity for certain metals, these can be targeted even when present in complex matrices and stabilized in biominerals (e.g., Pb in PbS, as per Staicu et al., 2020). Biominerals have high chemical stability often times exceeding greatly their chemical counterparts due to the contribution of their biological matrix (Konhauser and Riding, 2012). Some minerals (e.g., BaSO_4_) are classified as critical raw materials (CRM) due to their scarcity and economical/geostrategic importance. As such, the quest for metals and minerals needed to decarbonize the economy will stimulate the reevaluation of metal-rich wastes in the framework of circular economy (Staicu and Stolz, 2021).

This Research Topic comprises 5 original research articles and presents recent progress in the study of microbial biominerals exploring fundamental and applied aspects. Included are contributions on biogenic S^0^, twisted stalk formation, microbial diversity and biomineralization in metal-polluted mine waters, calcite biomineralization, and the economics of recovering biogenic magnetite. The paper of Cron et al. investigates the role of extracellular polymeric substance (EPS) in the formation and preservation of elemental sulfur, S^0^, biominerals produced by sulfur-oxidizing bacteria. Elemental sulfur organomineralization is documented in this paper using an environmental sample (a *Sulfurovum*-rich biofilm in the Frasassi Cave System, Italy).

Iron is essential for microbial metabolism (e.g, chemolithotrophic energy generation, regulatory proteins), however its processing can lead to detrimental biominerals for the host cell. The study by Koeksoy et al. looks into the underlying genetic and physiological mechanisms of twisted stalk formation (unique bacterial extracellular organo-metallic structures containing Fe(III) oxyhydroxides produced by Fe(II)-oxidizing bacteria). The paper presents a candidate gene cluster for the biosynthesis and secretion of the stalk organic matrix that was identified with a trait based analyses of a pan-genome comprising 16 Zetaproteobacteria isolate genomes.

The microbial diversity in metal-polluted mine waters is a key factor in the natural attenuation of industrial pollution. Paganin et al. propose a complex analysis including microbiological, mineralogical and geochemical data to study the indigenous sulfate-reducing bacteria (SRB) involved in metal biomineralization occurring in Iglesiente and Arburese districts (SW Sardinia, Italy). Interestingly, the most abundant genera found in the samples analyses in this study did not belong to the traditional SRB groups (i.e., *Rahnella, Acinetobacter*) indicative of site-specificity of natural microbial communities. The bio-precipitation process using selected cultures with polluted river water showed high removal of Zn (>97%) in the form of biogenic ZnS with tubular morphology.

An important research area focuses on the difference between biominerals and their non-biological counterparts. Zhao et al. employed a facultative anaerobic bacterium, *Enterobacter ludwigii* SYB1, to document the hydrochemistry, mineral crystallization, and cell surface characteristics of biomineralization. Their findings identified carbonate anhydrase and ammonia production as major factors influencing the alkalinity and saturation of the closed biosystem forming calcite, monohydrocalcite (MHC), and dypingite (hydrated magnesium carbonate mineral). Circular economy attempts to recover and reuse part of the “waste” products resulted from production cycles and microbes could be used to extract valuable minerals. In this issue, Correa et al. assessed the commercial viability of industrial production of magnetotactic bacteria (MTB)-derived nanomagnets. Biogenic magnetite is considered to possess superior characteristics and to entail a cleaner production in relation to synthetic nanoparticles, therefore the bioproduction costs are justifiably higher than chemical manufacturing.

Microbial biominerals are key components of the natural biogeochemical cycles of metals, but are also important in anthropogenic settings (industrial infrastructure, wastewater treatment) and industrial-contaminated sites. Their formation is often considered beneficial in terms of environmental depollution. Some future directions of research involving microbial biominerals should focus on (i) elucidation of their unknown biological functions with relevance for fundamental and applied knowledge, (ii) their recovery in the framework of the circular economy paradigm shift, and (iii) decontamination of ever-increasing sites affected by past and contemporary industrial activities.

## Author Contributions

All authors listed have made a substantial, direct, and intellectual contribution to the work and approved it for publication.

## Funding

LS acknowledges National Science Centre (NCN), Grant No. 2017/26/D/NZ1/00408. EH acknowledges H2020 ERA-MIN 2 funding for the BaCLEM project (Bio-assisted Closed loop recycling of E-Mobility Metals from waste PCBs and Li-Ion Batteries). CA acknowledges grant NIH R01 GM137139.

## Conflict of Interest

The authors declare that the research was conducted in the absence of any commercial or financial relationships that could be construed as a potential conflict of interest.

## Publisher's Note

All claims expressed in this article are solely those of the authors and do not necessarily represent those of their affiliated organizations, or those of the publisher, the editors and the reviewers. Any product that may be evaluated in this article, or claim that may be made by its manufacturer, is not guaranteed or endorsed by the publisher.
